# Characteristics of office workers who benefit most from interventions for preventing neck and low back pain: a moderation analysis

**DOI:** 10.1097/PR9.0000000000001014

**Published:** 2022-05-23

**Authors:** Nipaporn Akkarakittichoke, Mark P. Jensen, Andrea K. Newman, Pooriput Waongenngarm, Prawit Janwantanakul

**Affiliations:** a Inter-Department Program of Biomedical Sciences, Faculty of Graduate School, Chulalongkorn University, Bangkok, Thailand; b Department of Rehabilitation Medicine, University of Washington, Seattle, WA, USA; c Faculty of Health Science Technology, HRH Princess Chulabhorn College of Medical Science, Chulabhorn Royal Academy, Bangkok, Thailand; d Department of Physical Therapy, Faculty of Allied Health Sciences, Chulalongkorn University, Bangkok, Thailand

**Keywords:** Active break, Postural shift, Moderator, Neck pain, Low back pain, Sedentary worker

## Abstract

**Introduction:**

Neck and low back pain are significant health problem in sedentary office workers. Active break and postural shift interventions has been proved to reduce the incidence of new onset of both neck and low back pain.

**Objectives:**

To identify variables that moderate the effects of active breaks and postural shift interventions on the development of neck and low back pain in office workers.

**Methods:**

Using data from a 3-arm (active break, postural shift, and control group) cluster randomized controlled trial (N = 193), we evaluated the moderating effects of age, job position, education level, sex, perceived psychological work demands, number of working hours, and using a chair with lumbar support on the benefits of 2 interventions designed to prevent the development of neck and low back pain in office workers. Moderation analyses were conducted using the Hayes PROCESS macro, with post hoc Johnson–Neyman techniques and logistic regressions.

**Results:**

Significant interactions between intervention groups and 3 moderators assessed at baseline emerged. For the prevention of neck pain, the effect of the active break intervention was moderated by the number of working hours and the effect of the postural shift intervention was moderated by the level of perceived psychological work demands and the number of working hours. For the prevention of low back pain, the effect of postural shift intervention was moderated by having or not having a chair with lumbar support.

**Conclusions:**

The study findings can be used to help determine who might benefit the most from 2 treatments that can reduce the risk of developing neck and low back pain in sedentary workers and may also help us to understand the mechanisms underlying the benefits of these interventions.

## Abstract 2

Number of working hours, work-related psychological demands, and use of lumbar support moderate the beneficial effects of preventive interventions for neck and low back pain.

## 1. Introduction

Neck and low back pain are common in sedentary office workers. One-year prevalence rates for neck and low back pain among office workers have been shown to range from 42% to 69%^1,16^ and 31% to 51%,^2,39^ respectively. Although improvement of neck and low back symptoms can occur,^22,28^ the 1-year recurrence rate of neck and low back pain has been shown to range from 23% to 69%.^9,12^ Moreover, 17% and 27% of office workers who report a new onset of neck and low back pain report that these pain problems become chronic, respectively.^38^ Given that chronic pain represents a leading cause of disability worldwide^17^ and continues to be a major economic burden to society,^10^ identifying effective strategies to prevent neck and low back pain is of high importance. Moreover, identifying the individuals who would benefit the most from preventive treatments could improve the effectiveness and efficiency of treatment because this information could be used to inform better individual-treatment matching.

There is evidence supporting the effectiveness of exercise programs for reducing the risk of new episodes of neck pain^13^ and that exercise combined with education reduces the risk of subsequent episodes of low back pain.^41^ Research has also found that an intervention to increase daily walking steps reduced onset neck pain in high-risk office workers.^40^ Recently, we found that 2 interventions—one an active break and the second a postural shift intervention—reduced the incidence of new onset of both neck and low back pain.^45^ Both interventions were delivered by a custom-designed apparatus and were aimed to minimize seat discomfort by providing (1) recommendations for taking breaks (active break intervention) or (2) making postural shifts during prolonged sitting (postural shift intervention). However, in the primary outcome article, we did not conduct analyses to identify the moderators of treatment effects. Such research is important for being able to determine who might benefit the most from these interventions.

A systematic review found that the variables with strong evidence for moderating response to low back pain treatments (including cognitive-behavioral therapy, acupuncture, and manipulation combined with exercise) were age (younger participants may gain more benefits), employment status (those who were employed benefited more), occupation type (those in sedentary occupations benefited more), back pain severity (those who have worse initial back pain status benefited more), opioid medication use (those reporting less opioid use benefited more), treatment expectation (those with a greater positive expectations benefited more), and education level (those with greater than 10 years of schooling benefited more).^18^ Potential moderators with weaker evidence included sex, baseline psychological distress, baseline pain intensity, and baseline disability level.^18^ Another study reported that individuals with low back pain with high levels of fear avoidance were more likely to benefit from an educational booklet and an exercise program than were those with lower baseline levels of fear avoidance.^25^

To the best of our knowledge, no research has sought to identify the moderators of strategies to prevent neck and low back pain in office workers. To address this knowledge gap, here we used data from a completed randomized clinical trial^45^ to evaluate the moderation effects of different variables on the impact of the active break and postural shift interventions on the development of neck and low back pain. Given previous research and theory, we hypothesized that an active break intervention and postural shift approach would be more likely to reduce the risk of developing neck and low back pain in (1) younger participants than older participants, (2) general office workers than office managers, (3) participants with higher than a bachelor's degree than a bachelor's degree or lower, (4) female than male workers, and (5) those endorsing more psychological work demands than those endorsing fewer psychological work demands. In exploratory analyses, we also evaluated the potential moderating effects of a number of variables that have not yet been examined in previous research. Based on the available evidence regarding the predictors of neck or low back pain, we included baseline measures of chair height adjustability and the number of working hours as potential moderators of onset of neck pain^7,34^ and baseline measures of years of work experience, whether or not the participant engages in continuous standing for >2 hours/d, having a job that requires frequent trunk forward bending, and whether or not the worker's chair has lumbar support^23^ as potential moderators for the effects of the active treatments on the development of low back pain.

## 2. Materials and methods

### 2.1. Participants and procedures

The data for the analyses presented here came from a trial comparing the effects of active break and postural shift interventions designed to reduce the incidence of neck and low back pain, relative to no treatment, in office workers.^45^ Both the active break and postural shift interventions used a custom-designed apparatus, which consisted of 3 components: (1) a seat pad, (2) a processor, and (3) a smartphone application. The seat pad collected sitting behavior data, including the duration of any sitting breaks and the number of postural shifts. Data were stored in the processor and were used to compute recommended break durations and the number of postural shifts for each individual. For those in the active break intervention, instructions to take breaks were sent from the processor to the smartphone application. For those in the postural shift intervention, recommended postural shifts were induced by the apparatus gradually pumping air into various parts of the seat pad placed underneath a participant's buttocks. Commands to operate the seat pad were sent from the processor to the seat pad through a cord that connected them.

The study was a 3-arm, parallel group, cluster randomized controlled trial conducted using a convenience sample of 193 office workers who were at risk of developing nonspecific neck or low back pain. Details about the trial are available in the previous publication.^45^ The protocol was registered with the Thai Clinical Trials Registry (https://www.thaiclinicaltrials.org) (TCTR20190111002) and approved by the Chulalongkorn University Human Ethics Committee. Two eligibility criteria were adjusted subsequent to trial registration: (1) age (from 23–45 to 23–55 years) and (2) time needed to be pain free (from 12 to 6 months).

### 2.2. Descriptive variables

Baseline descriptive data were collected using a questionnaire assessing age, sex, height and weight (from which BMI was computed), education level, frequency of exercise, and smoking status. Work-related characteristics included job position, number of working hours, years of work experience, time per day spent using a computer, average number of hours/day of continuous standing and continuous sitting, whether work involved frequently engaging in 5 specific activities (ie, head extensions, head flexions, head rotations, trunk bending, and trunk rotations), and having or not having regular rest breaks. The questionnaire also asked participants to note different aspects of the ergonomics of their workstations and their work environments (ie, whether the desk height, computer screen level, position of a keyboard and mouse were suitable and whether or not the office had a comfortable air temperature, level of light brightness, and was well-ventilated).

Baseline neck and low back discomforts were assessed using the Borg CR-10 scale, which asked participants to rate the amount of perceived discomfort they felt in the past year in their neck and low back on 0 to 10 scales, with 0 = “no discomfort” and 10 = “extreme discomfort.”^4^ Discomfort was defined as the nonpainful but still uncomfortable feelings and sensations, such as tension, fatigue, or tremors,^19^ assessed using a body chart based on the modified Nordic questionnaire.^27^

### 2.3. Criterion variables: development of neck or low back pain

The 2 criterion variables in the current secondary analyses were new onset of nonspecific neck or low back pain, with or without radiation and without a disease identified as the underlying cause of the complaints *at the first episode of pain* during the 12-month follow-up period using a monthly diary.^5,26^ To assess this, the participants answered the question, “Have you experienced any neck or low back pain lasting >24 hours during the past month?” If they answered “yes,” a follow-up question about pain intensity measured using a visual analogue scale was asked, and the presence of weakness or numbness in the upper or lower limb was assessed. Those who (1) answered “yes” to the first question,^14^ (2) reported pain intensity >30 mm on a100-mm visual analogue scale,^42^ and (3) had no weakness or numbness in the upper or lower limbs (ie, to exclude symptoms more likely to be due to a serious medical conditions of neck or low back pain) were identified as cases.

### 2.4. Potential moderators

The potential moderators were classified into confirmatory or exploratory moderator variables. Moderators included in the confirmatory analyses were those for which we had a specific a priori hypothesis, based on previous evidence. Moderators classified as exploratory were those that have not yet been evaluated or for which a specific theory or mechanism is lacking.

The Descriptive Variables section above described the 4 confirmatory moderators (age, sex, job position, and education level). The fifth confirmatory variable was the level of psychological work demands. This variable was assessed using the 12-item Psychological Work Demands subscale of the Thai version of the Job Content Questionnaire.^35^ The internal consistency of this scale in the current sample was adequate (Cronbach alpha = 0.69).

Exploratory moderators of the neck pain onset included chair adjustability and number of working hours.^7,34^ Exploratory moderators of the low back pain onset included years of work experience and whether or not the participant (1) engages in continuous standing for >2 hours/day vs ≤2 hours/day, (2) describes themselves as needing to bend their trunk forward often during the work day, and (3) has a chair with lumbar support.^23^

### 2.5. Statistical analysis

Descriptive statistics were computed to describe the sample. All analyses followed an intention-to-treat approach (ie, last observation carried forward method). To estimate the associations between the potential moderators and the subsequent onset of nonspecific neck and low back pain in a cohort of office workers, analyses included the Hayes PROCESS 4.0 macro (model 1) in SPSS version 25 with 5000 bootstrapped samples (IBM SPSS; Preacher & Hayes, 2008). The analyses examined the interactions between the proposed moderators (ie, age, sex, job position, education level, and level of psychological work demands) and the treatment groups (active break, postural shift, and control) on the onset of neck and low back pain criterion variables (dichotomous). During March 2020, the COVID-19 outbreak occurred in Thailand. At the time, a majority of the study participants (68%) were required to work from home and did not bring the custom-designed apparatus or placebo foam pad home with them. Thus, status of working from home (yes/no) during the study was included as a covariate. Moreover, we controlled for the preintervention discomfort level on the dependent variables by using them as covariates to account for individual response tendencies and within-person variability. Treatment condition (ie, intervention groups and control group) was entered as 2 dummy coded variables. Potential moderators were also entered (1 per model tested), as was the Group × Moderator interaction.

To describe any significant moderations found, post hoc analysis was used including pairwise comparisons (ie, active break vs control group, postural shift vs control group, and active break vs postural shift) through PROCESS model 1 with the Johnson–Neyman technique and logistic regression analysis (an alpha level of 0.05). All statistical analyses were performed using SPSS for Windows version 25.0 (SPSS Inc, Chicago, IL). The regression models were constructed using the PROCESS macro version 4.0 (Andrew F. Hayes) for SPSS. Statistical significance was set at the 5% level and 95% CI.

## 3. Results

### 3.1. Sample description and general results of the moderator analyses

The trial spanned June 2019 to November 2020. The final sample included 193 office workers with a mean age of 33.8 (SD = 6.3) years. Most of the office workers were women (76%). Additional details regarding the demographic characteristics of the study participants are presented in Table 1.

**Table 1:** Descriptive characteristics of the study participants.

Characteristic	Active break (n = 47)	Postural shift (n = 46)	Control (n = 100)	*P*
Demographic characteristics				
Age (y)	31.6 (6.1)	35.0 (7.7)	34.1 (5.3)	0.008*
Sex: Female (%)	33 (70.2)	35 (74.5)	79 (79.0)	NS
BMI	21.3 (2.3)	22.3 (2.3)	21.0 (2.0)	0.002*
Education: higher than a bachelor's degree (%)	44 (95.7)	45 (95.7)	95 (95)	NS
Smoking status: No (%)	38 (80.9)	42 (91.3)	91 (91)	NS
Exercise frequency in the past 12 mo: Regularly (%)	7 (14.9)	10 (21.8)	22 (22.0	NS
Job position: Manager level (%)	45 (95.7)	44 (95.7)	95 (95)	NS
Baseline neck discomfort measured by the Borg CR-10 scale	1.5 (1.2)	1.3 (1.1)	1.9 (1.6)	NS
Baseline low back discomfort measured by the Borg CR-10 scale	2.9 (2.2)	2.2 (2.2)	3.7 (2.4)	0.001*
Work-related characteristics				
Number of working hours (per day)	8.0 (1.3)	8.7 (1.3)	7.8 (0.8)	NS
Year of work experience (y)	6.9 (4.3)	10.8 (5.3)	9.1 (4.8)	<0.001*
Chair adjustability: Yes (%)	46 (97.9)	43 (93.5)	64 (64)	<0.0001*
Continuous standing > 2 hours: No (%)	43 (91.5)	45 (97.8)	94 (94)	NS
Having or not having lumbar support: Yes (%)	20 (54.1)	16 (39.0)	43 (43)	NS
Often bending trunk forward: No (%)	36 (76.6)	37 (80.4)	78 (78)	NS
Psychosocial characteristics				
Psychological job demands	30.8 (4.4)	32.5 (4.2)	33.2 (4.4)	0.009*

* P < 0.05.

Three moderators, 1 from the list of confirmatory moderators and 2 from the list of exploratory moderators, emerged as significant. Table 2 presents the results of all tests of confirmatory and exploratory moderations.

**Table 2:** Results of analysis of preventive treatment (ie, active break and postural shift) effects.

Moderator	Onset of neck pain					Onset of low back pain				
	Estimate	SE	Effect size *(f*^*2*^*)*	95% CI	*P*	Estimate	SE	Effect size (*f*^*2*^)	95% CI	*P*
Active break vs postural shift vs control (n = 193)										
Confirmatory moderators										
Age	0.07	0.04	0.11	−0.01 to 0.16	0.096	0.01	0.05	0.05	−0.78 to 0.1	0.840
Sex	−0.38	0.64	0.11	−1.13 to 1.23	0.929	14.43	711.13	0.06	−1379.3 to 1408.2	0.984
Job position	−0.87	0.59	0.11	−2.04 to 0.31	0.147	1.05	0.70	0.05	−0.32 to 2.41	0.136
Education	−0.55	0.96	0.10	−2.44 to 1.34	0.568	14.81	633.57	0.04	−1226.9 to 1256.6	0.981
Level of work psychological demands	−0.12	0.06	0.14	-0.25 to −0.05	0.048*	-0.00	0.63	0.07	−0.13 to 0.12	0.650
Exploratory moderators for neck pain										
Chair adjustability	1.16	0.74	0.11	−0.29 to 2.61	0.119					
Number of working hours	−0.69	0.34	0.17	−1.35 to −0.03	0.040*					
Exploratory moderators for low back pain										
Years of work experience						0.05	0.05	0.06	−0.44 to 0.13	0.323
Continuous standing						-0.76	1.29	0.04	−3.29 to 1.76	0.554
Not having lumbar support						−1.97	0.66	0.09	−3.27 to −0.67	0.003*
Often bending trunk forward						-0.38	0.65	0.04	−1.65 to 0.89	0.556
Active break vs control (n = 147)										
Level of work psychological demands	−0.73	0.11	0.14	−0.29 to 0.14	0.051	0.05	0.15	0.09	−0.23 to 0.34	0.703
Number of working hours	−1.39	0.55	0.26	−2.47 to −0.32	0.010*					
Not having lumbar support		-0.64	1.45	0.19	−3.48 to 2.21	0.635				
Postural shift vs control (n = 146)										
Level of work psychological demands	−0.146	0.07	0.14	−0.28 to −0.01	0.030*	-0.03	0.06	0.05	−0.14 to 0.08	0.650
Number of working hours	−0.69	0.32	0.11	−1.33 to −0.05	0.033*					
Not having lumbar support						−2.09	0.69	0.11	−3.44 to −0.74	0.003*
Active break vs postural shift (n = 93)										
Level of work psychological demands	−1.69	0.14	0.05	−0.45 to 0.11	0.241	-0.11	0.17	0.02	−0.44 to 0.23	0.532
Number of working hours	0.12	0.53	0.09	−0.93 to 1.17	0.820					
Not having lumbar support						−3.14	1.81	0.00	−6.69 to 0.41	0.095

* P < 0.05.

### 3.2. Number of working hours/day as a moderator

**Figure 1.**
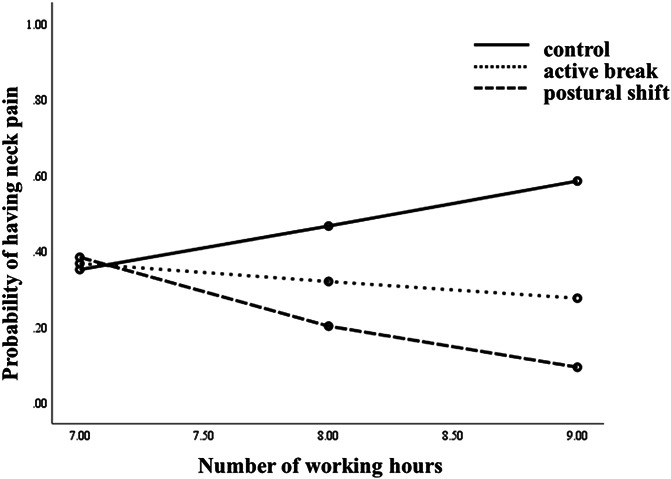
Illustration of the Group × Working Hours moderation effect on the probability of having neck pain.

**Figure 2.**
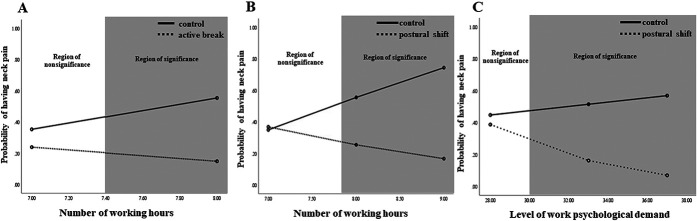
Significant moderations of the differences in the treatment effect and region of significance difference in (A) active break vs control groups and (B and C) postural shift vs control groups on the onset of neck pain.

The analysis for the onset of neck pain revealed a significant Treatment Group X Number of Working Hours/Day interaction (B = −0.69, *SE* =0.34, *R*^*2*^ = 0.33, *P* =0.040; Fig. 1). Post hoc comparisons indicated a difference between the active break vs control group (B = −0.39, *SE* = 0.55, *R*^*2*^ = 0.44, *P* = 0.010) and postural shift vs control group (B = −0.69, *SE* = 0.32, *R*^*2*^ = 0.37, *P* = 0.033); participants who reported working more hours/day at baseline gained more benefits from both interventions, compared with those in the control group. Johnson–Neyman results indicated that participants in the active break group who reported working >7.4 hours/day, and participants in the postural shift group who reported working >7.8 hours/day, were more likely to benefit from the interventions to prevent the onset of neck pain in comparison to the control group, who also worked these hours at baseline (Fig. 2A and B). Logistic regressions analyzing within-treatment conditions indicated that the number of hours of work per day was a significant predictor of neck pain for the active break (B = −0.6, *SE* = 0.28, *OR* = 0.55, 95% CI 0.32–0.95, *R*^*2*^ = 0.18, *P* = 0.032) and control groups (B = 0.81, *SE* = 0.31, *OR* = 2.26, 95% CI 1.23–4.23, *R*^*2*^ = 0.12, *P* = 0.008; Table 3), indicating that more hours of work per day were associated with increased neck pain.

**Table 3:** Within-group simple slopes for significant tests of moderation (ie, simple effects).

Moderator	Active break (n = 47)				Postural shift (n = 46)				Control (n = 100)			
	B	SE	β	*P*	B	SE	β	*P*	B	SE	β	*P*
Nonspecific neck pain												
Level of work psychological demands	0.03	0.89	0.97	0.784	−0.09	−0.89	0.91	0.297	0.11	0.05	1.12	0.022*
Number of working hours	−0.60	0.28	0.55	0.032*	−0.49	0.39	0.62	0.220	0.81	0.31	2.26	0.008*
Nonspecific low back pain												
Not having lumbar support	0.93	1.27	2.53	0.465	−1.09	0.87	0.34	0.290	1.86	0.47	6.39	< 0.001*

* P < 0.05.

### 3.3. Psychological work demands as a moderator

**Figure 3.**
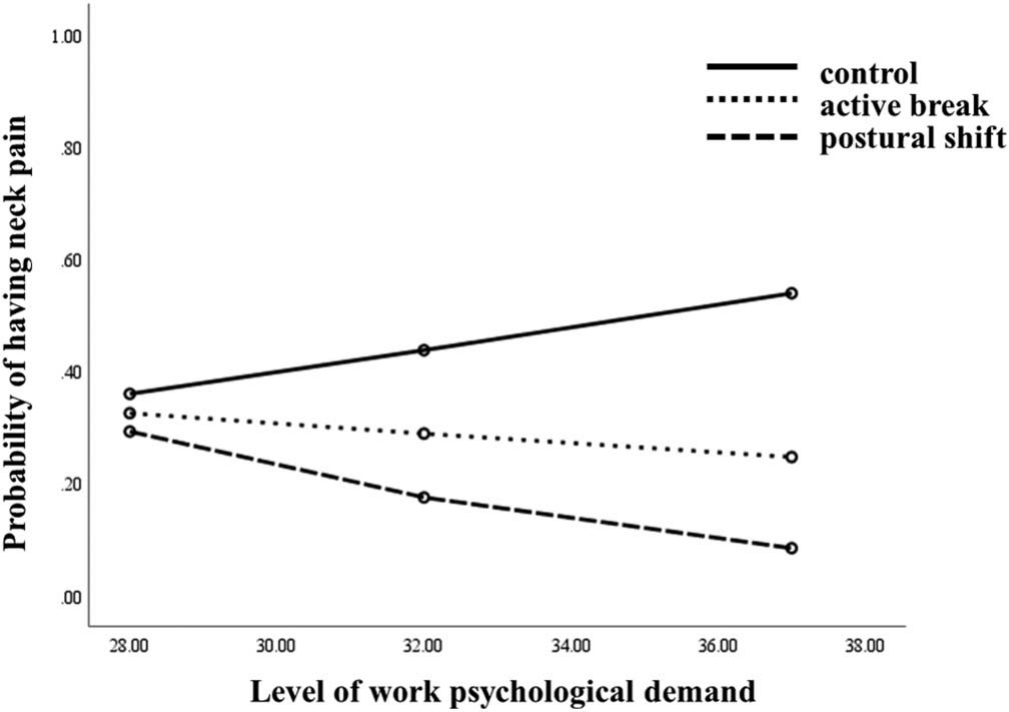
Significant moderations of the differences in group condition effects (ie, active break, postural shift, and control groups) on neck pain.

The moderation analysis for the onset of neck pain revealed a significant Treatment Group X Psychological Work Demands interaction (B = −0.12, *SE* = 0.06, *R*^*2*^ = 0.32, *P* = 0.048; Fig. 3). Post hoc pairwise comparisons indicate a significant difference between the postural shift and control group (B = −0.15, *SE* = 0.07, *R*^*2*^ = 0.37, *P* = 0.030); participants who endorsed higher levels of psychological work demands at baseline and who were assigned to the postural shift group were less likely to develop neck pain than those randomized to the control group. Johnson–Neyman results indicated that participants with a level of work psychological demands >30.6 were less likely to develop neck pain with the postural shift condition compared with those in the control condition with this same level of perceived work demands (Fig. 2C).

The perceived level of psychological work demands evidenced a nonsignificant trend to be a moderator (B = −0.73, *SE* = 0.11, *R*^*2*^ = 0.38, *P* = 0.051) for the active break vs control group and active break vs postural shift group (B = −1.69, *SE* = 0.14, *R*^*2*^ = 0.22, *P* = 0.241) with respect to the development of neck pain. Logistic regressions suggested that the level of work psychological demands was a significant predictor of neck pain only for the control condition (B = 0.11, *SE* = 0.05, *OR* = 1.12 95% CI 1.01–1.24, *R*^*2*^ = 0.08, *P* = 0.022; Table 3), in which more psychological demands were associated with increased neck pain.

### 3.4. Having or not having lumbar support as a moderator

**Figure 4.**
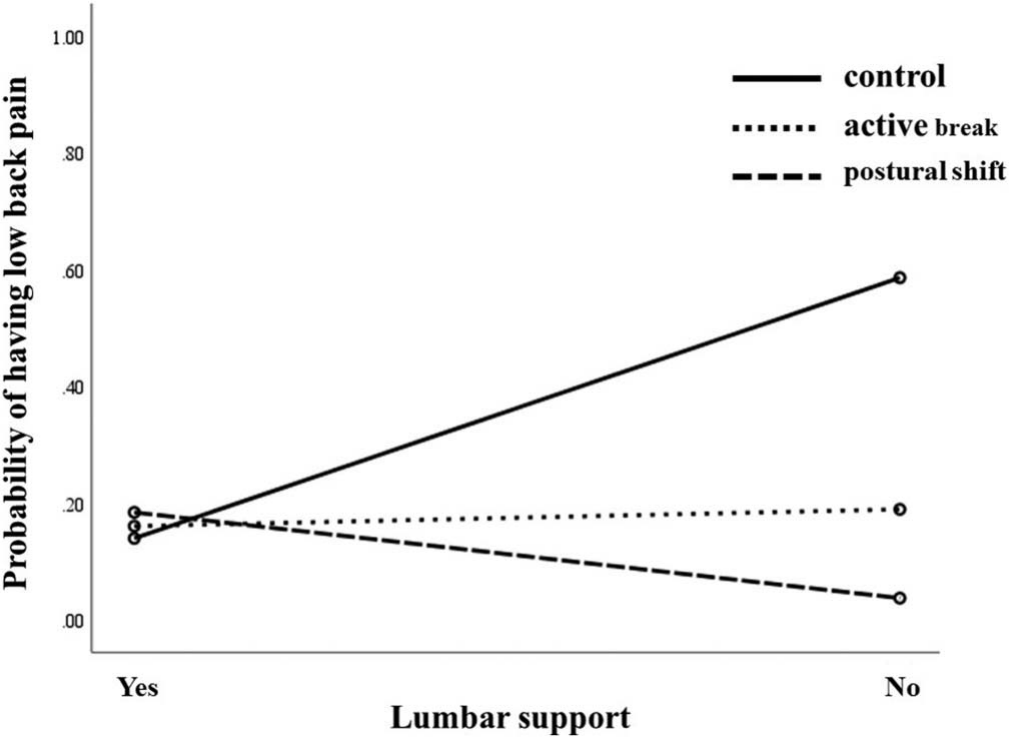
Significant moderations of the differences in group condition effects (ie, active break, postural shift, and control groups) on low back pain.

The analysis for the onset of low back pain revealed a significant Treatment Group X Lumbar Support interaction (B = −1.97, *SE* = 0.66, *R*^*2*^ = 0.37, *P* = 0.003; Fig. 4). Post hoc comparisons indicated a significant difference between the postural shift and control group (B = −2.09, *SE* = 0.69, *R*^*2*^ = 0.42, *P* = 0.003); participants who reported not having lumbar support gained more benefits from the postural shift intervention than those in the control group. However, having or not having lumbar support was not found to predict any benefit for the active break vs control group (B = −0.64, *SE* = 1.45, *R*^*2*^ = 0.42, *P* = 0.635) or active break vs postural shift group (B = −3.14, *SE* = 1.81, *R*^*2*^ = 0.15, *P* = 0.095). Logistic regressions suggested that having or not having lumbar support was a significant predictor of low back pain for the control condition (B = 1.86, *SE* = 0.47, *OR* = 6.39, 95% CI 2.52–16.20, *R*^*2*^ = 0.23, *P* < 0.001; Table 3); not having lumbar support was associated with increased low back pain.

## 4. Discussion

This study sought to identify workers more or less likely to achieve benefits from interventions designed to reduce the risk of developing neck and low back pain. With respect to neck pain, we found that workers who worked more hours/day gained more benefits from both the active break and postural shift interventions than from the control condition and that workers who endorsed higher levels of psychological work demands gained more benefits from the postural shift intervention. The only moderator found with respect to preventing low back pain was that workers who had no lumbar support gained more benefits from the postural shift intervention than those in the control group. These findings have important implications for understanding who might benefit the most from these treatments, as well as for understanding the potential mechanisms underlying those benefits.

### 4.1. Moderators of prevention treatments and possible mechanisms

We found that the effects of the active break and postural shift interventions on neck pain risk were moderated by the number of working hours/day. Previous research has shown that both prolonged sitting and working time increase the risk of developing neck pain.^7,36^ The present findings suggest that the 2 interventions studied may mitigate the negative effects of longer working hours. Given the previous findings that increasing daily walking steps reduces the onset neck pain in office workers,^40^ it seems possible that the beneficial effects of taking breaks on neck pain could be mediated by increases in walking steps that could occur during those breaks. With respect to the postural shift intervention, changing sitting postures has been found to result in changes in cervicothoracic muscle activity.^8^ It is therefore possible that changing sitting postures may impose alternating activity in the neck and shoulder muscles, resulting in the alleviation of postural discomfort during prolonged sitting and consequently reducing the risk of developing neck pain.

We also found a significant moderation effect for the level of psychological work demands on the benefits of the postural shift intervention, and *a nonsignificant* trend (*P* = 0.051) for this same moderation effect for the active break intervention. Consistent with this finding, Gurung and colleagues^18^ identified psychological distress as a potential moderator of the benefits of cognitive-behavioral therapy on back pain–related disability. Other researchers have found that negative mood and emotions, cognitive functioning, and pain behavior are all related to the onset of neck pain and other musculoskeletal conditions.^3,30^ We can envision 2 possible mechanisms for the moderation effect we observed. First, psychological stress could induce negative physical effects (eg, muscle tension^47^ or postural discomfort^44^), which may then be mitigated by position shifts or by taking breaks. Second, taking active breaks or postural shifts might directly reduce psychological stress. Consistent with this idea, previous research has found that breaks can reduce or prevent stress and help to facilitate recovery from early symptoms of work-related mental and physical fatigue.^37^ However, research is needed to test psychological stress as a potential mediator of the beneficial effects of these treatments.

The moderation effect of lumbar support on the beneficial effects of the postural shift intervention for low back pain is consistent with previous research showing that lumbar support helps to prevent the development of low back pain.^23^ Prolonged sitting with poor posture (ie, reduced lumbar–lordotic curve) might increase strain on the back of the spine, consequently increasing the risk of hyperflexion injury to the disk and ligament.^24,31^ The negative effects of poor sitting posture can be minimized by using lumbar support, which contributes to maintaining the natural lordotic curvature.^29^ Regularly performing postural shifts while sitting may also decrease static trunk muscle activity,^8^ thus reducing trunk muscle fatigue and delaying the onset of low back discomfort.^32,33^ The concept that sitting with poor posture can contribute to exacerbations of low back symptom is controversial.^6,11^ Thus, the association between sitting posture and low back pain remains tenuous. Additional research to clarify the roles of sitting posture, lumbar support, and postural shifts in the development of low back pain is warranted.

### 4.2. Variables not showing a significant moderation effect

The hypothesized moderators that did not emerge as significant included age, job position, education level, and sex. The exploratory moderators that did not emerge as significant were having an adjustable chair, years of employment, standing for more than 2 hours in a typical work day, and needing to bend one's trunk forward frequently during work. These null findings have a number of possible explanations. First, it is possible that these factors moderate the effects of treatments that were not examined here. For example, the hypothesized moderators have been shown in previous research to moderate the effects of passive treatments such as acupuncture (ie, age and sex)^46^ and spinal manipulation (ie, low back pain duration and baseline pain intensity).^15^ By contrast, we examined the effects of active interventions, which may have different moderators. A second possible reason may be related to differences in the study samples. Hayden and colleagues described that having or not having heavy physical demands at work, using or not using medications, and BMI level moderated the effects of exercise treatment,^20^ and Underwood and colleagues found that age and employment status moderated the effects of a cognitive-behavioral intervention in individuals with low back pain.^43^ However, in the current study, the participants were office workers who were not currently reporting any neck or back pain. Additional research is needed to determine the extent to which moderators influence outcome across different samples of individuals and different types of treatment.

### 4.3. Implications for understanding mechanisms

The fact that the interventions studied here were moderated by 2 factors that seem to be biomechanical in nature (ie, longer work day hours and lack of lumbar support) is consistent with the idea that the interventions studied reduce the risk of developing neck and back pain by reducing biomechanical stress. This idea could be tested by including objective measures of biomechanical stress (eg, EMG activity) during treatment and then conducting mediation analyses^21^ to test biomechanical stress as a potential mediator. It is also possible that having more work hours could affect pain through its effects on psychosocial stress. Research to examine both types of stress as potential mediators is warranted.

### 4.4. Study strengths and limitations

A major strength of this study is a conservative definition of neck and low back pain (ie, pain that is moderate in intensity^42^ and lasts for at least 24 hours^14^) as our measure of pain onset. It is noteworthy that the results might have differed had we used a less conservative operational definition of pain onset.

The study has 4 primary limitations. First, we did not control for multiple testing. It is possible that some of the significant effects found emerged because of chance alone. The analyses conducted here should be repeated in other samples. Second, there are potential moderators identified in previous research that we did not evaluate here. Future research should examine these moderators. Third, the sample size for these analyses was not determined a priori because they are secondary analysis using data from a completed study. It is possible that significant moderation effects might have gone undetected. However, we did compute the effect sizes (*f*^*2*^*)* of the moderation effects tested, which may be used to determine the sample size by future researchers. Finally, the study was conducted using otherwise healthy 23 to 55 years old office workers who were pain free at the time of study enrollment. Therefore, generalization of the findings to other populations should be made with caution.

## 5. Conclusions

The findings provide new and important information regarding the role that several factors play as moderators of the effects of active break and postural shift interventions. We found significant moderating roles for (1) the number of working hours/day for the benefits of the active break intervention and (2) the number of working hours/day and perceived psychological work demands for the benefits of the postural shift intervention. The role of not having lumbar support was a moderator of the effect of the postural shift intervention in the development of low back pain. These findings can be used to identify those workers who are most likely to benefit from the interventions and provide preliminary findings that could inform future studies to evaluate the mechanisms that underlie the beneficial effects of these treatments.

## Disclosures

The authors have no conflicts of interest to declare.
